# Physical Exercise in Guillain-Barré Syndrome: A Scoping Review

**DOI:** 10.3390/jcm14082655

**Published:** 2025-04-12

**Authors:** Pawel Kiper, Manon Chevrot, Julie Godart, Błażej Cieślik, Aleksandra Kiper, Martina Regazzetti, Roberto Meroni

**Affiliations:** 1 Healthcare Innovation Technology Lab, IRCCS San Camillo Hospital, 30126 Venice, Italy; 2 Department of Health, LUNEX International University of Health Exercise and Sports, L-4671 Differdange, Luxembourg; 3 Doctoral School, University of Rzeszów, 35-310 Rzeszów, Poland; 4 Luxembourg Health & Sport Sciences Research Institute ASBL, L-4671 Differdange, Luxembourg

**Keywords:** physical activity, fatigue, strength, functional independence, physiotherapy, treatment, Guillain-Barré syndrome

## Abstract

**Background**: Guillain-Barré Syndrome (GBS) is a rare post-infectious, immune-mediated inflammatory disorder of the peripheral nervous system that can manifest in multiple distinct forms. It significantly impacts patients’ quality of life, causing both short-term and long-term impairments, including reduced strength, respiratory deficits, functional limitations, decreased endurance, and increased fatigue. This scoping review aimed to assess the impact of physical activity on strength, functional independence, and fatigue in patients with GBS, as well as to identify effective types of physical activity for rehabilitation programs. **Methods**: A literature search was conducted in March 2024 and updated in June 2024 across PubMed, Embase, Cochrane, Scopus, and Web of Science using predefined inclusion and exclusion criteria. We included full-text, peer-reviewed articles written in English, French, Polish, or Italian that focused on strength, fatigue, and functional independence within the context of physical exercise. **Results**: A total of 1021 papers were eligible for screening, and after the screening process, 16 papers were included in this review. **Conclusions**: Physical exercise may enhance strength, reduce fatigue, and promote functional independence in GBS. Recommended interventions often include muscle strengthening, functional training, and endurance exercises. Larger, high-quality studies and further research into chronic fatigue mechanisms are needed to refine long-term rehabilitation strategies.

## 1. Introduction

Guillain-Barré syndrome (GBS) is an umbrella term for a group of rare, post-infectious immune-mediated inflammatory disorders of the peripheral nervous system, characterized by a range of clinical manifestations and variants. The syndrome occurs with an incidence of 0.8 to 1.9 cases per 100,000 person-years [1,2]. Among its various forms, Acute Inflammatory Demyelinating Polyradiculoneuropathy (AIDP) and Acute Motor Axonal Neuropathy (AMAN) are the most common, presenting distinct patterns of sensorimotor and motor impairments, respectively [3,4]. Additionally, rarer variants such as Miller Fisher Syndrome (MFS), characterized by ataxia, areflexia, and ophthalmoplegia, and Bickerstaff’s Brainstem Encephalitis (BBE) expand the clinical spectrum of GBS [5].

Approximately two-thirds of GBS patients experience symptoms of respiratory or gastrointestinal infections prior to onset, with the pathogen *Campylobacter jejuni* implicated in about 30% of cases [3]. The disease mechanism is thought to involve an aberrant immune response where antibodies mistakenly attack components of the peripheral nerves, a process potentially triggered by a molecular mimicry of the lipooligosaccharides found on the *Campylobacter jejuni* membrane. This immune cross-reactivity can lead to significant nerve damage and dysfunction [6].

Patients with GBS present with rapidly progressing motor and/or sensory impairments symmetrically affecting the lower and upper limbs, with the most common symptoms being numbness, tingling, and weakness progressing to paralysis; in some cases, respiratory problems can be observed [7]. Hyporeflexia or areflexia is often present [1]. Pain frequently precedes the onset of weakness and can be severe [8]. Symptoms evolve during the progressive phase, the critical phase begins two weeks after the symptoms’ onset, and maximal weakness is reached within four weeks; the plateau phase is seen before recovery starts and can last from seven days to six months [3]. Despite achieving significant neurological recovery, many of them will remain severely fatigued [9]. Fatigue is an important concern in GBS patients and is associated with a negative impact on their quality of life (QoL) [10]. Its causes and management are complex and poorly understood [8].

Treatment for GBS primarily involves immunotherapy, specifically plasma exchange (PE) or intravenous immunoglobulin (IVIg), which have both demonstrated significant efficacy [11]. Due to the potential for long-term residual impairments, regular monitoring and rehabilitation are essential to mitigate and recover from these effects [7]. Beyond specific medical treatments, physical activity also plays a crucial role in the overall management of GBS. Regular exercise is not only beneficial for preventing conditions like cancer, cardiovascular diseases, and type 2 diabetes but also promotes functional ability, bone density, and muscle strength [12]. Specifically, in the context of neuropathic conditions, exercise training has been shown to improve nerve function and reduce neuropathic pain and other sensory symptoms in patients with peripheral neuropathy [13].

The body of literature exploring the utilization of physical activity for this patient group is limited. In 2016, Arsenault et al. [14] conducted a systematic review incorporating seven intervention studies. They concluded that due to the lack of high-quality literature, it is challenging to draw definitive conclusions regarding the impact of exercise interventions on physical outcomes in patients with GBS [14]. Nearly a decade after its publication, this study remains the sole review that employs a systematic search method on this subject. Consequently, the question of which type of exercise is effective for treating GBS continues to be unresolved.

Therefore, the aims of this scoping review were to assess whether the use of physical exercise can reduce fatigue, improve muscle strength, and enhance the ability to perform daily activities independently in patients with GBS. Additionally, this review aimed to identify the types of physical exercise interventions used in the treatment of GBS, focusing on their implementation and outcomes related to fatigue, strength, and functional independence.

## 2. Materials and Methods

### 2.1. Study Design

This study was designed as a scoping review and followed the JBI methodology for a scoping review [15]. Guidelines from the Preferred Reporting Items for Systematic Reviews and Meta-Analysis extension for scoping reviews (PRISMA—ScR) [16] were used to guide the elaboration of the review and convey results. A study protocol was registered a priori on https://osf.io/428bx/ (accessed on 9 April 2025).

Following the Population, Concept, and Context (PCC) framework, inclusion criteria were determined. Included studies had to implement some type of physical exercise in a rehabilitation setting aimed toward either muscle strength, fatigue, or functional independence in patients with diagnosed GBS or one of its variants. For the papers to be included, the rehabilitation process had to include physical exercise supervised by a physiotherapist. Different rehabilitation strategies were considered, and with “physical exercise”, we refer to physical therapy (functional training, endurance training, and gait training), physical activity (aerobic exercise, strengthening exercise, mobility, and range of motion exercise), group or individual physical activity, and personalized or conventional physical activity. The intervention had to take place in a clinical or home-based setting. We focused the context of the review on the influence of physical activity on strength, fatigue, and functional independence as the three main outcomes. These outcomes had to be assessed using reliable tools, and the results had to be clearly reported.

Primary and secondary studies, qualitative, quantitative, and mixed-method research were considered with no regard to the publication date and whether they were written in English, French, Italian, or Polish. Included articles had to exist as full-text peer-reviewed articles and focus on at least one of the three outcomes previously stated. When no abstract was available for articles, they were automatically screened through a full-text reading. Abstracts, conference papers, short communications, protocols, letters, reviews, and research not focusing on the outcomes of interest were not included.

### 2.2. Information Sources and Search Strategies

The literature search was run in March 2024 on PubMed, Cochrane, Embase, Scopus, and Web of Science and updated in June 2024. The final search strategies for each database are shown in Appendix A (Table A1), listing the keywords applied during the search and the Boolean operators ‘AND’ and ‘OR’, as well as the MESH terms that were used to refine the search. After retrieving the available literature search, the results were uploaded to Rayyan platform [17] to allow for duplicate removal as well as results screening.

Once the duplicates were removed, two authors (M.C. and J.G.) screened, independently and blinded, the articles through titles and abstracts in Rayyan software [17]. During this phase, any disagreements between reviewers regarding the eligibility of a study were resolved through discussion or by consulting a third reviewer (B.C.). This step ensured that only the most relevant studies were included in the final review. The screening of the results followed the PRISMA-ScR guidelines [16]. After the title and abstract screening was completed, only articles selected for full-text reading remained, including articles where no abstracts were available but from which the title contained elements of the PCC. A PRISMA flow diagram was used to illustrate the selection process, detailing the number of studies included and excluded at each stage, along with reasons for exclusion.

### 2.3. Data Extraction and Synthesis

To provide an overview of the most relevant information from the selected studies, we utilized a data extraction table. Established a priori during the development of the review protocol, this table highlights key points from each study, including the title, authors, year of publication, country, study design, aims, sample size/population characteristics, intervention/type of physical exercise, outcome measures, and key findings. Additionally, the studies were categorized into three groups based on their designs: RCTs, observational studies, and case series/case studies.

## 3. Results

### 3.1. Search Results

**Figure 1:**
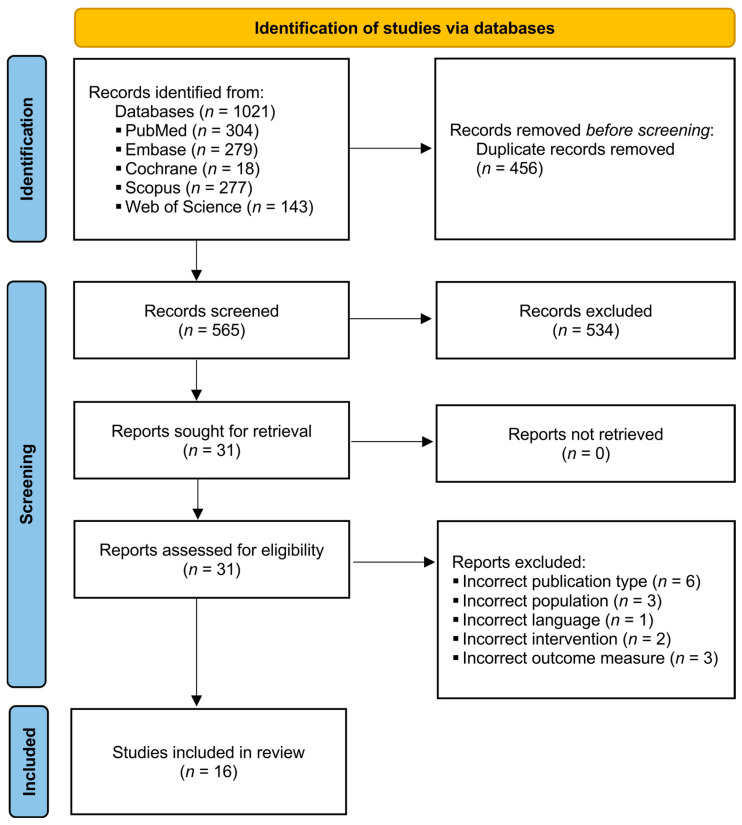
Flow diagram.

Following a search of the literature, 565 articles were identified after duplicate removal (Figure 1). After full text reading, we included sixteen papers of which seven (43.75%) were case reports [18,19,20,21,22,23,24], two (12.5%) were case studies [25,26], two (12.5%) were randomized controlled trials [27,28], two (12.5%) were case series [29,30], two (12.5%) were prospective studies [31,32], and one (6.25%) was a cohort study [33]. The number of participants varied between one and seventy-nine, with a greater prevalence of studies with one participant due to the number of case reports and case studies. In total, 181 participants were assessed in the included papers. All participants were affected by Guillain-Barré Syndrome or one of its variants; specifically, 14 participants presented with AIDP, 137 with GBS, 7 with Acute Motor/Motor–Sensory Axonal Neuropathy (AMSAN), 2 with MFS, 15 with CIDP, and 6 with OH.

### 3.2. Narrative Synthesis of the Results

The data of the selected articles are reported in the data extraction table (Table 1). Articles described various training programs including up to 20 weeks of rehabilitation training and 12 months of home-exercise programs. Overall, programs consisted of muscle strengthening, aerobic training, functional rehabilitation, gait training, respiratory exercises, balance training, or range of motion mobilizations. Ten studies implemented dynamic exercise or muscle strengthening exercises [18,19,20,21,22,23,24,26,27,28], eleven implemented functional training, mobility, ADL’s training, and gait training [18,19,20,21,22,23,24,26,27,28,30], seven implemented endurance training/bicycle training [18,25,26,27,28,29,32], six implemented range of motion exercises [18,19,20,21,23,24], and seven implemented home-exercise programs [18,21,24,27,28,30,31]. Out of the sixteen studies, two did not provide details on the type of rehabilitation offered to the participants [31,33]. Regarding the outcomes of interest, thirteen studies demonstrated an improvement in muscle strength [18,19,20,21,22,23,24,25,26,28,31,32,33], fourteen studies showed an improvement in functional independence [18,19,20,21,22,23,26,27,28,29,30,31,32,33], and five studies showed a decrease in fatigue [26,28,29,30,32]. For functional independence, the papers demonstrated improvement between the start and the end of the rehabilitation programs. The RCT from Khan et al. [27] reported statistically significant changes in its described outcome as the intervention showed a significant improvement in the total Functional Independence Measure (FIM) scores in both groups, with a 68% increase for the intervention group and a 32% increase for the control group (*p* < 0.003). The prospective study from Garssen et al. [32] also showed significant changes from baseline for functional independence (*p* < 0.01). Uz et al.’s [33] cohort study demonstrated significant changes between admission and discharge in functional independence and ambulation (*p* = 0.0001). The case series from Janssen et al. [30] revealed a 10% improvement, not statistically significant, whereas Bussmann et al.’s [29] case series demonstrated a significant improvement in functional independence as well as fitness.

Considering the papers evaluating strength changes, they demonstrated overall improvement in patients’ strength. Garssen et al. [32] also demonstrated a statistically significant improvement from baseline in strength (*p* < 0.01), as well as Uz et al. [33] (*p* = 0.000). El Mhandi et al.’s [31] study demonstrated an increase in strength that was statistically significant (*p* < 0.01 for the first 6 months follow-up; *p* < 0.05 between 6 and 18 months follow-up).

Only five papers assessed and discussed the influence of physical activity on fatigue, and these studies reported a decrease in fatigue among the patients evaluated. Only the study from Garssen et al. [32] and the study from Bussmann et al. [29] described statistically significant changes in fatigue compared to baseline measurement (*p* < 0.01 and *p* < 0.05, respectively). Janssen et al. [30] showed a 10% decrease in fatigue without statistical significance. Uz et al. [33] did not find significant changes in fatigue compared to strength and functional independence.

### 3.3. Recommendations for Physical Activity in GBS Rehabilitation

Based on the findings of the included studies, the following table provides an overview of recommended physical activity interventions for patients with GBS (Table 2). These recommendations are derived from the reported interventions and their associated statistically significant outcomes. This table summarizes the types of interventions applied, the number of studies incorporating them, their reported frequency and duration, and their statistical significance where applicable. Muscle strengthening and functional training were the most frequently implemented interventions, while endurance training, ROM and stretching exercises, respiratory exercises, and proprioceptive training appeared less frequently in the selected studies. From the 16 selected papers, the studies from Uz et al. [33] and El Mhandi et al. [31] did not provide specifics on the rehabilitation programs they offered to participants.

## 4. Discussion

In this scoping review, the current literature investigating the effects of physical activity on strength, fatigue, and functional independence in GBS patients was summarized. Overall, our review shows that physical exercise is a favorable intervention for improvements in GBS patients and should be considered an important aspect of GBS treatment. Our wide inclusion criteria allowed the provision of results unrelated to gender, age, or ethnic background. The observed positive impact of physical activity on strength, fatigue, and functional independence in GBS patients is consistent with the results of a previous systematic review [14]. It is also in line with a more recent review, from Sulli et al. [34], which highlighted a correlation between physical therapy interventions and enhancement in GBS patients’ well-being.

Improvements in muscle strength and functional independence were observed in the majority of the appraised articles; however, the link between decreased fatigue and physical activity is less apparent. According to Bussmann et al. [29], there is a reduction in the self-reported fatigue in GBS patients after exercise therapy, but this decrease does not seem significantly influenced by an improved physical fitness. Different types of fatigue exist; in the acute phase of GBS, peripheral fatigue is the main contributor, while in the chronic phase, the experienced fatigue might be influenced by other psychosocial factors [35]. Physical activity correlates with psychological well-being [36] and improves sleep quality and depressive/anxiety symptoms [37]. We could hypothesize that findings of reduction in fatigue might arise from these physical activity benefits, but a study from Ranjani et al. [9] showed fatigue was independent of the severity of weakness, depression, and sleep disturbance. Future research on the causes of fatigue in chronic GBS should be conducted.

In the case report by Connors et al. [22], evaluated patients received education on energy conservation while performing ADLs. Physiotherapists should include this education time in their GBS rehabilitation plan since fatigue plays an important role in the ability of patients to follow a physiotherapy session. In the reviewed studies, exercise dosages were different. Physiotherapists should adjust their approach based on the patient’s current condition.

The studies we reviewed used different types of physical exercise interventions. In seven studies, the intervention consisted of a combination of strengthening, functional, and ROM exercises with no aerobic training [19,20,21,22,23,24,30]. Endurance training alone was prescribed in three studies [25,29,32]. Four studies combined muscle strengthening, balance, functional exercises, and aerobic training [18,26,27,28]. Two studies did not provide details of their intervention, which limits the interpretation of the results [31,33]. All studies observed a positive influence of physical exercise on patients’ physical outcomes, despite the intervention type. Physiotherapists should consider combining all types of physical activity in their rehabilitation program to provide optimal rehabilitation. Studies with different groups, each assigned to one of these interventions, would be interesting to compare their effects and find the more effective approach to improve physical outcomes in GBS patients.

Our review included two RCTs, and both compared an outpatient rehabilitation program versus a home-based program but with different aims. Shah et al. [28] aimed to investigate the difference in the effects between a supervised exercise program and an unsupervised home program. Both resulted in functional independence, fatigue, and muscle strength improvement. The supervised program showed better enhancement; however, the home-exercise program should not be neglected for the continuity of treatment in the chronic phase. Guidelines from van Doorn et al. [8] advise continuing home and/or outpatient physiotherapy for more than six months since functions can continue to improve for many months after the acute phase of the disease. The elaboration of a universal, specific, and detailed home-exercise program for GBS patients, tailored to each individual’s needs, would provide patients with a more efficient treatment.

Khan et al. [27] aimed to compare the effects of a high- and a low-intensity program on functional independence. High-intensity intervention seemed more efficient compared to low-intensity intervention. This finding could be due to the lack of supervision of the home-based program and not the difference in intensity.

Out of the sixteen included studies, only one prospective observational study addressed the topic of intervention side effects. Five patients out of thirty experienced mild and transient side effects such as muscle cramps, burning sensation, pain, and paresthesia in the leg [32]. In practice, physiotherapists should inform patients about these potential side effects to prevent panic if any of these symptoms occur. Further studies should be conducted to investigate the possible side effects of physical exercise in GBS.

One of the case reports included in this review described the effect of a rehabilitation program in a patient with postinfectious COVID-19-associated AIDP. The patient was also diagnosed with a right ankle fracture, which prevented weight-bearing for 6 weeks [22]. This unexpected event interfered with the original rehabilitation plan, and the results might have been different without this restriction. GBS patients can present with comorbidities, limiting some interventions and affecting their recovery outcomes. Physiotherapists must be prepared for unexpected events and consider comorbidities during the rehabilitation process to provide the more efficient and safer treatment.

### 4.1. Clinical Recommendations and Future Directions

The findings emphasize the importance of incorporating multiple exercise modalities to optimize recovery in patients with GBS. Muscle strengthening at moderate intensity (2–3 weekly sessions, 2–3 sets of 10–15 repetitions) has repeatedly been linked to significant improvements in force generation and functional independence, with some evidence suggesting that these gains can persist for at least six months when sessions are adequately supervised. Functional training, including gait retraining and balance exercises, likewise yields measurable enhancements in mobility and self-care activities, which can remain evident at a twelve-month follow-up if structured support is maintained. Endurance exercises, such as stationary cycling or aerobics, reduce fatigue and bolster general conditioning; however, the benefits may wane after prolonged intervals without continued participation, underscoring the need for sustained engagement. In addition, range-of-motion activities—though not always accompanied by direct statistical significance—serve an essential preventative function by mitigating contractures and preserving flexibility, particularly in the early stages of recovery. Proprioceptive interventions and respiratory-focused exercises further augment these gains in selected populations, although additional data are required to firmly establish their long-term impact and delineate their optimal dosage.

Further investigations are needed to refine the frequency, intensity, and sequencing of exercises to accommodate GBS’s clinical variability, particularly among patients with differing fatigue and respiratory profiles. Larger-scale randomized trials using standardized outcome measures and extended follow-up are also indicated to determine the durability of therapeutic benefits and inform optimal post-discharge care. Future research should also explore the physiological mechanisms underlying improvements associated with specific exercise modalities, thus providing deeper insight into their effectiveness. In parallel, advances such as tele-rehabilitation and robot-assisted gait training show promise for enhancing adherence and outcomes, especially when continuous supervision is impractical. Comparative research on purely home-based versus hybrid or fully supervised models will likewise clarify resource allocation and patient-centric strategies. These efforts will guide the development of individualized, sustainable interventions that maximize function and quality of life for individuals affected by GBS.

### 4.2. Study Limitations

Our review has some limitations. Due to the rarity of the disease, there is a lack of studies on the topic. The majority of papers are low-evidence studies. There are very few RCTs available, with a prevalence of case reports or case series. These are uncontrolled study designs with an increased risk of bias [38]. Our results, therefore, need to be interpreted with caution and cannot be generalized to the population. Nevertheless, it is important to take them into account. The task force of the European Academy of Neurology/Peripheral Nerve Society Guideline on the diagnosis and treatment of GBS considers physiotherapy and other rehabilitation treatments to be important in both the acute and chronic phases, despite the paucity of strong evidence [8]. The intervention length was not the same in every included paper. In some studies, patients had different GBS variants. Our selected studies included patients from different phases: acute, subacute, and chronic. These differences in parameters can create confounding factors and may affect our findings.

## 5. Conclusions

Physical exercise shows measurable benefits for strength, fatigue reduction, and functional independence among individuals with GBS, with muscle strengthening, functional training, and endurance exercises representing core components of effective rehabilitation. Preliminary evidence suggests that sustained engagement and structured supervision can extend these gains beyond six months. However, heterogeneity in clinical presentations—particularly regarding fatigue severity and respiratory status—necessitates tailored approaches. Larger, high-quality randomized controlled trials with extended follow-up are warranted to refine optimal exercise parameters (e.g., intensity, frequency, and sequencing) and to evaluate emerging modalities such as tele-rehabilitation, robot-assisted gait training, and proprioceptive interventions. In parallel, elucidating the underlying causes of severe fatigue in the chronic phase holds promise for guiding targeted interventions. Ultimately, a multifaceted, patient-centered exercise program that is continuously monitored and adjusted may offer the greatest potential for maximizing long-term outcomes and quality of life in GBS.

## Figures and Tables

**Table 1:** Data extraction table.

Author (s), Year	Sample Size/Population Characteristics	Intervention/Type of Physical Exercise	Dose	Outcome Measures	Key Findings
RCTs (*n* = 2)					
Shah et al. (2022) [28]	Sixteen participants with a mean age of 33 (±13) years in the experimental group and 47 (±17) years in the control group.	Intervention group: Strengthening exercises, endurance training, and gait training. Control group: Maintenance exercises and self-management education.	A 12-week supervised program (60 min sessions, 2–3 times per week).	BI, MRC, VAS, FSS, WHOQoL–BREF	Both groups improved in functional independence, fatigue, and muscle strength, with greater gains in the EG. Pain decreased only in the EG, and the largest quality of life improvement was in the WHOQoL-BREF environment domain.
Khan et al. (2011) [27]	Seventy-nine participants (31 females), with a mean age of 54.9 ± 17.1 years.	Intervention group: Strengthening exercises, endurance training, gait training, functional training for everyday activities, driving, and return to work. Control group: Maintenance exercises (walking, stretching) and education.	A 12-week program (3 sessions per week, 1 h each) for the intervention group.	FIM, WHOQoL-BREF, DASS, PIPP	Patients in the high-intensity program showed significantly higher FIM scores with large effect sizes compared to the low-intensity program. No changes were observed in DASS and WHOQoL-BREF, except in the PIPP “relationship” subscale.
**Observational studies (*n* = 3)**					
Uz et al. (2023) [33]	Twenty-four participants (mean age 47.29 ± 16.2 years; 9 females).	Inpatient physical therapy during hospitalization, followed by prospective evaluations after discharge.	Not specified	FIM, FAS, Hughes scale, 6MWT, FSS, MRC	Significant improvements were observed in the FIM, Hughes scale, FAS, 6MWT, and MRC scores. Negative correlations were found between age and the FAS, 6MWT, and MRC scores at the first year of follow-up.
El Mhandi et al. (2007) [31]	Six patients (mean age 48.0 ± 15.2 years; 3 females).	Adapted individualized rehabilitation program, including muscular reinforcement and active mobilizations.	Inpatient rehabilitation for 3–4 weeks, followed by 4–10 weeks of outpatient rehabilitation and a home-exercise program.	MMT, Dynamometry, FIM	Isometric and isokinetic strength increased significantly during the first 6 months, with slower gains between 6 and 18 months. Functional independence improved markedly in the first 6 months and remained at maximal levels in subsequent follow-ups.
Garssen et al. (2004) [32]	Thirty participants (20 patients, 14 females).	Bicycle exercise training program, including warm-up, cycling, and cool-down sessions with progressive intensity adjustments.	A 12-week program with 3 supervised sessions per week, progressing from a 70% to 90% maximum heart rate.	FSS, RAM, Scale, GBSDS, RHS, SF-36, muscle strength	Significant reductions in fatigue severity and improvements in psychological well-being and quality of life. Enhanced physical fitness, increased muscle strength, and better GBS disability scores were also observed.
**Case series/case reports (*n* = 11)**					
Gawande et al. (2024) [23]	A 20-year-old female with GBS, with bilateral limb weakness, severe lower limb pain, motor dysfunction, and severe difficulty walking.	Early hospital-based rehabilitation, including PROM exercises, passive contract–relax stretching, PNF techniques, isometric and dynamic strengthening, breathing exercises, and supine-to-sit transition training.	Daily sessions with PROM, PNF, strengthening, and breathing exercises.	MMT, Hughes scale, NPRS, FIS, TGS	Increased upper and lower limb muscle strength, improved functional independence, and reduced pain levels.
Almeida et al. (2023) [26]	A 58-year-old male with GBS and prior COVID-19 infection, presenting with progressive lower limb weakness, fatigue, and dyspnea.	Individualized, functional goal-oriented treatment, including muscle strengthening, balance, aerobic, functional, and swallowing training, with patient and family education.	A 6-week program (5 h/day, 5 days/week).	FACIT-FS, 6MWT, BESTest, FIM, FOIS, MRC-MMT	Improvements in balance, mobility, and functional status alongside enhanced respiratory and peripheral muscle strength. Fatigue levels and dyspnea were reduced.
Boob et al. (2022) [18]	A 22-year-old male with MFS, presenting with bilateral symmetrical limb weakness, slurred speech, and swallowing difficulties.	Frenkel’s exercises, positioning, ROM exercises, muscle strengthening, aerobic training, coordination and balance exercises, supported standing and walking, and faradic stimulation for facial muscles.	A 6-week progressive daily exercise program, followed by a home-exercise program incorporating all phases.	MMT, Hughes scale, FIM	Significant functional improvement, enhanced recovery trajectory, better quality of life, and long-term benefits, including increased muscle strength and improved performance in ADLs.
Connors et al. (2022) [22]	A 61-year-old male with postinfectious COVID-19-associated AIDP, presenting with progressive bilateral limb weakness.	Comprehensive PT and OT approach including individual and group exercise sessions, motor coordination, cognitive tasks, and mobility training, with education.	Daily 30 min sessions (5–6/day, Monday–Friday), with a 30-min group session on Saturday.	ROM, MMT, Dynamometer Modified FIM, ADLs	Improved lower limb function (strength, ROM, coordination), evolution in cognitive function, and reduction in functional impairment.
Nagore et al. (2022) [21]	A 62-year-old male diagnosed with ADIP, presenting with bilateral weakness in the upper and lower limbs and kyphotic posture.	Respiratory exercises, passive mobilizations, ankle pumps, static and dynamic exercises, assisted active ROM for upper and lower limbs, stretching (hamstrings/quadriceps), pelvic bridging, resistance training, and sit-to-stand training.	Daily sessions, progressing from passive and static exercises to active, resistance, and gait training, with a home-exercise program and follow-up after 15 days.	BBS, FIM, MMT, ROM	Improved muscle strength, ROM, functional capacity, endurance, overall quality of life, and ADLs.
Tanaka et al. (2022) [20]	Female in her 20s diagnosed with demyelinating type of GBS, presenting with distal extremity numbness, weakness, dysphagia, and limited ROM.	Muscle strengthening, ROM training, hand dexterity, self-care exercises, postural training, gait training, wheelchair mobility, static and dynamic balance exercises, and functional activities like eating, grooming, dressing, and cooking.	Therapy progressed from bedside exercises to supervised gait training and functional activities, with gradual integration of assistive devices and outdoor mobility training over 20 weeks.	MMT, Ankles ROM, 6MWT, 10MWT, FIM, Grip strength, STEF	Improved upper and lower limb muscle strength, ankle ROM, gait, functional independence, and endurance.
Vishnuram et al. (2022) [19]	A 35-year-old male with AMSAN, hyponatremia, and alcoholic hepatitis, presenting with paraparesis, diaphragm weakness, and bilateral lower limb weakness with sensory deficits.	Upper limb muscle strengthening, lower limb assisted and passive ROM exercises, DVT prophylaxis, joint compression, positioning, incentive spirometry, breathing exercises, prolonged supported sitting, assisted transfers, bed mobility, reaching exercises, and standing with support.	Thirty-minute sessions, 3 times per day for 4 weeks, with progressive intensity and focus on muscle strengthening, mobility, and functional independence.	Vital signs, MRC, GBSDS	Gradual normalization of vital signs, improved hip and knee muscle strength, and a decrease in GBSDS scores.
Janssen et al. (2018) [30]	Case series (*n* = 7) of patients aged 48–77 diagnosed with CIDP.	Otago Home-Exercise Program with a personalized exercise plan, including strength and balance exercises and a walking routine.	Six weeks (3 times per week), complemented by at least 30 min of walking weekly.	BBS, 10MWT, FSS, EQ5D-5L	Exercise program positively impacted walking speed and balance, with significant improvements in 10MWT and BBS scores. A change in FSS and EQ5D-5L was not statistically significant.
Akinoğlu et al. (2016) [24]	A 66-year-old male with MFS, presenting with sensory loss in extremities, impaired vision, gait, and postural control.	Muscle strengthening, flexibility training, balance and coordination exercises, functional training (sitting, standing, gait, stairs), mobilizations (soles, foot joints, cervical spine), sensory education, and mirror feedback for posture and balance.	Physical therapy delivered over 5 years, supplemented by a home-exercise program.	Muscle strength and shortness, one leg standing, BBS, stairs climbing, gait speed	Decreased lower extremity muscle shortness, increased upper and lower limb muscle strength, improved one-foot standing, independent walking, stair climbing abilities, and increased BBS scores.
Bussmann et al. (2007) [29]	Case series (*n* = 20) with a median age of 49 years (14 females, 6 males).	Supervised cycle training sessions.	Three times/week for 12 weeks.	Muscular power, FSS, SF-36-physical, FIS, RHS, HAD, SF-36	Increased physical fitness, decreased fatigue, improved body mobility, and perceived functioning, with no significant relationship between fitness and other domains.
Pitetti et al. (1993) [25]	A 57-year-old male with GBS, presenting with bilateral lower limb weakness	Training on a Schwinn Air-Dyne ergometer, including warm-up, aerobic training, and cool-down.	A 16-week program with 30 min sessions, 3 days per week.	Peak work level, Ventilation, Isokinetic Strength	Improved cardiopulmonary capacity, lower limb muscle strength, total work capacity, and peak work level.

ADLs: Activities of Daily Living; ROM: range of motion; BBS: Berg Balance Scale; FIM: Functional Independence Measure; MMT: Manual Muscle Test; 6MWT: 6-Minute Walk Test; 10MWT: 10-Meter Walk Test; STEF: Simple Test for Evaluating Hand Function; RCT: randomized controlled trial; *n*: number of patients; Int: intervention group; WHOQoL-BREF: World Health Organization Quality of Life Scale; DASS: Depression Anxiety Stress Scale-21; PIPP: Perceived Impact of Problem Profile; AMSAN: Acute Motor Sensory Axonal Neuropathy; DVT: Deep Vein Thrombosis; GBSDS: Guillain-Barré Syndrome disability score; CIDP: Chronic Inflammatory Demyelinating Polyneuropathy; FSS: Fatigue Severity Score; RAM: Rotterdam Activity Monitor; FIS: Fatigue Impact Scale; HAD: Hospital Anxiety and Depression Scale; SF-36: Short Form-36 Health Questionnaire; FACIT-FS: Functional Assessment of Chronic Illness Therapy-Fatigue Subscale; FOIS: Functional Oral Intake Scale; PT: physical therapy; OT: Occupational Therapy; EG: experimental group; VAS: Visual Analog Scale of Pain; FAS: Functional Ambulation Scale; PNF: Proprioceptive Neuromuscular Facilitation; NPRS: Numeric Pain Rating Scale; TGS: Tone Grading System.

**Table 2:** Physical activity recommendations.

Exercise Category	Exercise Types	*n*	Recommended Dose	Significance	Long-Term Benefits	Comments
Muscle Strengthening	Static, Active/Dynamic, Resistance, Isotonic/Isometric/Isokinetic, Bridging, Rhythmic Stabilization	10 studies [18,19,20,21,22,23,24,26,27,28]	2–3x/week, 2–3 sets of 10–15 reps	Increased muscle strength, functional independence	Strength gains persisted at 6 months	Supervised exercise led to greater strength gains than home-based programs
Functional Training	Gait, Transfers, Sit-to-Stand, Postural and Balance Training, ADLs	studies [18,19,20,21,22,23,24,26,27,28,30]	Daily or 3–5x/week	Significant improvements in functional independence, gait stability, and mobility	Functional gains maintained at 12 months in structured rehab	Higher-intensity rehab had stronger effects than home programs
Endurance Training	Stationary Bike, UL/LL Cyclergometer, Aerobic Training	7 studies [18,25,26,27,28,29,32]	2–3x/week, 30 min per session	Significant reduction in fatigue, improvements in functional independence	Fatigue benefits declined after 12 months without continued training	Lower intensity initially recommended for severe fatigue cases
ROM and Stretching	Passive Mobilization, Active-Assisted ROM, Ankle Pumps, DVT Prevention, Static Stretching	6 studies [18,19,20,21,23,24]	2–3x/day, 10–20 reps per joint; 15–30 s holds for stretches	No direct statistical significance reported but supports flexibility, mobility, and functional recovery	No long-term data available	Important for early-phase rehab, prevents contractures, and enhances flexibility
Proprioceptive Training	PNF Techniques	2 studies [23,24]	3x/week	No statistically significant results reported	No long-term data available	May aid neuromuscular control, but more evidence is needed
Respiratory Exercises	Pursed Lip Breathing, Incentive Spirometry, Segmental Breathing Exercise	4 studies [18,19,21,23]	2–3x/day	No statistically significant results reported but beneficial for respiratory function and endurance	No long-term data available	Recommended for patients with respiratory impairment due to GBS

GBS: Guillain-Barré Syndrome; ADLs: Activities of Daily Living; ROM: range of motion; DVT: Deep Vein Thrombosis; PNF: Proprioceptive Neuromuscular Facilitation.

## Data Availability

Not applicable.

## Appendix A

**Table A1:** Final database search strategies.

Database	Final Search Phrase
**PubMed**	(“Guillain-Barre Syndrome”[MeSH Terms] OR “Guillain-Barre Syndrome” OR “GBS” OR “acute inflammatory demyelinating polyneuropathy” OR “acute polyradiculoneuropathy”) AND (“Exercise”[MeSH Terms] OR “Physical Therapy Modalities”[MeSH Terms] OR “physical exercise” OR “physical activity” OR “rehabilitation” OR “exercise therapy” OR “physical therapy” OR “physiotherapy” OR “strength training” OR “aerobic exercise” OR “mobility training”) AND (“Fatigue”[MeSH Terms] OR “Muscle Strength”[MeSH Terms] OR “Activities of Daily Living”[MeSH Terms] OR “fatigue” OR “muscle strength” OR “strength” OR “functional independence” OR “daily living activities” OR “quality of life” OR “mobility” OR “physical function”)
**Scopus**	(TITLE-ABS-KEY (“Guillain-Barre Syndrome”) OR TITLE-ABS-KEY (“GBS”) OR TITLE-ABS-KEY (“acute inflammatory demyelinating polyneuropathy”) OR TITLE-ABS-KEY (“acute polyradiculoneuropathy”)) AND (TITLE-ABS-KEY (“physical exercise”) OR TITLE-ABS-KEY (“physical activity”) OR TITLE-ABS-KEY (“rehabilitation”) OR TITLE-ABS-KEY (“exercise therapy”) OR TITLE-ABS-KEY (“physical therapy”) OR TITLE-ABS-KEY (“physiotherapy”) OR TITLE-ABS-KEY (“strength training”) OR TITLE-ABS-KEY (“aerobic exercise”) OR TITLE-ABS-KEY (“mobility training”)) AND (TITLE-ABS-KEY (“fatigue”) OR TITLE-ABS-KEY (“muscle strength”) OR TITLE-ABS-KEY (“strength”) OR TITLE-ABS-KEY (“functional independence”) OR TITLE-ABS-KEY (“daily living activities”) OR TITLE-ABS-KEY (“quality of life”) OR TITLE-ABS-KEY (“mobility”) OR TITLE-ABS-KEY (“physical function”))
**Web of Science**	TS = (“Guillain-Barre Syndrome” OR “GBS” OR “acute inflammatory demyelinating polyneuropathy” OR “acute polyradiculoneuropathy”) AND TS = (“Exercise” OR “Physical Therapy Modalities” OR “physical exercise” OR “physical activity” OR “rehabilitation” OR “exercise therapy” OR “physical therapy” OR “physiotherapy” OR “strength training” OR “aerobic exercise” OR “mobility training”) AND TS = (“Fatigue” OR “Muscle Strength” OR “Activities of Daily Living” OR “fatigue” OR “muscle strength” OR “strength” OR “functional independence” OR “daily living activities” OR “quality of life” OR “mobility” OR “physical function”)
**Embase**	(‘guillain-barre syndrome’:ti,ab,kw OR gbs:ti,ab,kw OR ‘acute inflammatory demyelinating polyneuropathy’:ti,ab,kw OR ‘acute polyradiculoneuropathy’:ti,ab,kw) AND (‘exercise’:ti,ab,kw OR ‘physical therapy modalities’:ti,ab,kw OR ‘physical exercise’:ti,ab,kw OR ‘physical activity’:ti,ab,kw OR ‘rehabilitation’:ti,ab,kw OR ‘exercise therapy’:ti,ab,kw OR ‘physical therapy’:ti,ab,kw OR ‘physiotherapy’:ti,ab,kw OR ‘strength training’:ti,ab,kw OR ‘aerobic exercise’:ti,ab,kw OR ‘mobility training’:ti,ab,kw) AND (‘fatigue’:ti,ab,kw OR ‘activities of daily living’:ti,ab,kw OR fatigue:ti,ab,kw OR ‘muscle strength’:ti,ab,kw OR strength:ti,ab,kw OR ‘functional independence’:ti,ab,kw OR ‘daily living activities’:ti,ab,kw OR ‘quality of life’:ti,ab,kw OR mobility:ti,ab,kw OR ‘physical function’:ti,ab,kw)
**Cochrane**	(“Guillain-Barre Syndrome” OR “GBS” OR “acute inflammatory demyelinating polyneuropathy” OR “acute polyradiculoneuropathy”) AND (“Exercise” OR “Physical Therapy” OR “Physical Therapy Modalities” OR “physical exercise” OR “physical activity” OR “rehabilitation” OR “exercise therapy” OR “physiotherapy” OR “strength training” OR “aerobic exercise” OR “mobility training”) AND (“Fatigue” OR “Muscle Strength” OR “Activities of Daily Living” OR “functional independence” OR “quality of life” OR “mobility” OR “physical function”)

ti, ab, kw: Search of Title field, Abstract field, Keyword field.
